# Watch the tone of your voice! An exploration of dehumanization of women by gender nonconformity based on tone of voice, occupation and appearance

**DOI:** 10.3389/fpsyg.2024.1387876

**Published:** 2024-05-15

**Authors:** Veysi Tanriverdi, Aydan Yurdagül, Ezgi Aze Tulum, Mustafa F. Ozbilgin

**Affiliations:** ^1^Department of Psychology, Bülent Ecevit University, Zonguldak, Türkiye; ^2^Brunel Business School, College of Business, Arts and Social Sciences, Brunel University London, London, United Kingdom

**Keywords:** voice tone, gender expectations, gender nonconformity, animalistic dehumanization, mechanistic dehumanization

## Abstract

Dehumanization refers to the act of likening others to objects or animals. This, in turn, mitigates feelings of conscience, guilt, and moral obligation in the face of behaviors such as violence, mistreatment, or discrimination against the dehumanized individuals. The aim of this study is to determine the extent of which women with mismatching vocal tone, occupation and appearance to their gender expectations are dehumanized by others. To achieve this, we conducted a between-groups factorial design experiment. In the experiment, participants looked at the photo and listened to the voice of a target woman with either a gender congruent or incongruent vocal tone, occupation, or appearance. Participants indicated the extent to which human attributes were appropriate for this individual. The results revealed that the main effects of vocal tone and occupation were significant for both mechanistic and animalistic dehumanization. A target woman with a mismatched vocal tone and occupation was more dehumanized compared to those with a matched vocal tone and occupation. However, the interaction effect of vocal tone, occupation type, and appearance was found to be significant only for mechanistic dehumanization. Our study provides evidence to recent concerns that women may experience dehumanization due to their vocal tone and occupation.

## Introduction

The BBC Worklife (2018) reported that women on television and radio have been altering their tone of voice in preference for deeper tones, emulating masculine tones, in the BBC recorded history. This simple news item led us to question the root causes of this change. Delving into the literature on the tone of voice (Andrews and Schmidt, 1997), we uncovered that the gender conformity and nonconformity of tone of voice has not been studied in terms of dehumanization of women at work.

Historically, deviating from gender norms or expectations has resulted in negative attitudes and reactions both in social life (Fiske and Stevens, 1993; Eagly and Wood, 2012) and in the workplace (Abele et al., 2016; Donnelly and Twenge, 2017). In order to avoid these negative consequences, both women and men have been performed their gender expectations that leading to the perpetuation of gender inequalities (see Butler, 1990 for performing gender). Therefore, studies demonstrating how gender incongruence affects both social and work life in terms of gender roles, gender expression, and voice tone, and how this negative impact can be mitigated, are of paramount importance. Additionally, determining how these variables interact with each other will contribute to taking more accurate steps towards eliminating gender inequalities and formulating more appropriate policies. This study investigates the impact of women’s voice tones, professions, and appearances on dehumanization. To achieve this goal, the study utilizes the two-dimensional dehumanization model proposed by Haslam (2006) and examines whether women with different combinations of gender incongruity experience mechanistic or animalistic dehumanization through experimental methods.

The following section delves into gender expectations in workplace. Subsequently, we analyze the gendered tone of voice, based on masculinity and femininity. We then investigate gender stereotypes and gender inequalities within the context of Turkey, and propose the study’s hypothesis. Defining and explaining our methodological approach, we proceed to present our findings and draw conclusions. The paper concludes by addressing limitations and offering recommendations for future research.

### Gender expectations in workplace

Traditional norms of masculinity and femininity are acquired through social learning processes, influencing the behaviors of individuals (Hemsing and Greaves, 2020) and contributing to the development of gender stereotypes, particularly in terms of competence and warmth (Eagly, 1987). These norms pertain to specific behaviors, expectations, and attributes that societies and cultures have constructed and accepted throughout history. Masculine roles encompass qualities expected from or attributed to men by society, while feminine roles represent attributes expected from or attributed to women (Eagly, 1987; Mahalik et al., 2005; Eagly and Wood, 2012). Therefore, women are traditionally expected to take on caregiving or emotional support roles within the family, while men are directed more towards outward, leadership roles (Eagly and Sczesny, 2019). In another word, expectations about possessing certain characteristics associated with gendered attributes profoundly affect career choices and can lead to gender discrimination, influences individuals’ occupation choices, promotion opportunities, leadership skills, and work relationships (Koenig and Eagly, 2014; Abele et al., 2016; Donnelly and Twenge, 2017). Consequently, men have typically occupied high-status roles, such as those in technology, science, leadership, and engineering, which are often associated with masculinity, whereas women have been more commonly found in low-status roles, such as domestic responsibilities, nursing, and early childhood education, which are typically associated with femininity (Eagly, 2013).

Masculinity is often associated with competence, while femininity is associated with warmth (Fiske et al., 2002). Consequently, gender expression, appearances, and clothing choices play crucial roles in the workplace, significantly impacting individuals’ professional identities. This is because gender expression is a critical determinant in assessing an individual’s masculinity or femininity (Bullough and Bullough, 1993, p. 312).

Gender expression is defined as the way individuals present themselves to others (Morrow and Messinger, 2006). Women are typically encouraged to adopt more feminine clothing styles, while men are expected to prefer masculine styles. For instance, men are often expected to wear dark-colored clothing and have short hair, while women are expected to opt for lighter clothing and have long or loose hairstyles (Koenig, 2018; Van Grootel et al., 2018). Therefore, these factors can influence how individuals are perceived in terms of competence and warmth, given the association of competence with masculinity and warmth with femininity. Research has shown that a masculine appearance positively affects the perceived competence not only of men but also of women (von Rennenkampff et al., 2003; Klatt et al., 2016). However, masculinity can be associated with the attribution of fewer human nature characteristics to women (Heflick et al., 2011), a phenomenon known as mechanistic dehumanization (Haslam, 2006). Furthermore, individuals are expected to conform to gender roles stereotypically, and deviations from these roles can lead to negative reactions (Jetten et al., 2013). For example, Men who pursue traditionally “feminine” careers are often ridiculed (Isacco and Morse, 2015), while women who pursue traditionally “masculine” careers may face discrimination and bias (Jetten et al., 2013). Therefore, women in occupations incongruent with female stereotypes may also be dehumanized in both mechanistic and animalistic ways.

### Gendered tone of voice

Women’s voices are often characterized by higher pitch and softer tones, aligning with societal expectations of femininity, whereas men’s voices tend to be deeper and resonate more, reflecting masculine ideals (Köylü, 2016). Examining the typical voice characteristics associated with gender reveals differences in fundamental acoustic components, particularly in frequency and formants. Lower-frequency voices are often perceived as more masculine, while higher-frequency voices are associated with femininity (Pisanski and Bryant, 2019). The larger larynx, longer vocal tracts, and lower-frequency voices commonly found in men are linked with masculine traits (Ohala, 1983, 1984). Therefore, tone of voice is a component cue for impression formation (Zimman, 2018), can influence the perception of attributes such as age, body size (see Puts et al., 2012; Pisanski and Bryant, 2019), attractiveness, intelligence and competency (Fraccaro et al., 2013; Hughes et al., 2014; Leongómez et al., 2014).

Research has indicated that, individuals with thin and sharp voices may be perceived as less trustworthy, less tense, and less emotional, while slower speakers might be viewed as less reliable, less credible, and less open (Apple et al., 1979). Despite women generally having higher-pitched voices, both women and men with voices that align with gender expectations are often considered attractive (Feinberg, 2008; Pisanski and Bryant, 2019). Another study found that lower voice pitch is associated with attributions of dominance and trustworthiness to the speaker (McAleer et al., 2014). Klofstad et al. (2012) demonstrated that participants were more likely to perceive a candidate as competent and trustworthy when speaking at a lower pitch, regardless of gender. Similarly, Borkowska and Pawlowski (2011) found that both men and women with lower-pitched voices were perceived as more dominant and trustworthy. Furthermore, Tsantani et al. (2016) found that lower-pitched voices were consistently perceived as more trustworthy, especially for female speakers. However, for dominance, while lower-pitched male voices were preferred, there was no significant difference for female speakers. Moreover, Oleszkiewicz et al. (2016) reported conflicting results regarding female voice pitch. They found that lower-pitched female voices were rated as more competent but less warm compared to higher-pitched voices, adding complexity to the understanding of voice pitch in impression formation. Additionally, there are no studies showing the effect of voice tone on mechanical and animalistic dehumanization. Overall, the evidence on the role of female voice pitch in impression formation is inconclusive and requires further investigation.

### Dehumanization of women

Dehumanization refers to a social process that leads to the perception of a group or an individual as lacking in humanity or the denial of their humanity (Smith, 2014). It has been studied in two primary forms, as defined by Haslam et al. (2005). Individuals to whom characteristics, such as curiosity, warmth, and possessing emotions were less attributed, were perceived as cold, passive, lifeless, and superficial, resembling machines/robots or objects. This dimension was termed “mechanistic dehumanization.” On the other hand, individuals to whom characteristics, such as politeness, open-mindedness, rationality (analytical thinking), linguistic skills, maturity, and moral sensitivity, were less attributed were perceived as uncultured, rude, irrational, immoral, and childlike. Since the reduced attribution of these characteristics implied likening humans to animals, this dimension was termed “animalistic dehumanization” (Haslam et al., 2007). Both forms of dehumanization are associated with negative outcomes among individuals and groups (Haslam and Loughnan, 2014), such as less altruistic behavior and greater acceptance of violence toward outgroups (Viki et al., 2013; Andrighetto et al., 2014; Ellemers, 2017). It can occur both consciously, supporting aggressive policies (Kteily et al., 2015; Jardina and Piston, 2016; Kteily and Bruneau, 2017), and unconsciously towards specific groups (Haslam and Stratemeyer, 2016). Therefore, it also can trigger potential discrimination even without explicit hostility or, at the very least, exacerbate it (Bruneau et al., 2020).

Dehumanization of women is often ascribed to gender stereotypes (Diekman and Goodfriend, 2006) that are fundamentally based on the dimensions of competence and warmth (Fiske et al., 2002). Competence relates to perceived abilities such as intelligence, skills, and effectiveness, while warmth relates to perceived intentions, including friendliness, helpfulness, and sincerity (Fiske et al., 2007). Throughout history, women have often been stripped of their personhood and reduced to their bodily and sexual functions (see Fredrickson and Roberts, 1997 for sexual objectification). This enduring gendered perspective has led to the attribution of warmth and emotionality, associated with relational orientation, to women, while agency and competence, linked with task orientation, have been predominantly ascribed to men (see Fiske et al., 2002; Cuddy et al., 2008 for the stereotype content model; see Gray et al., 2007 for mind perception theory). The dehumanization of women is often linked to their objectification (Loughnan et al., 2010). Women are often perceived as resembling animals (Vaes et al., 2011), objects (Bernard et al., 2012), or both (Rudman and Mescher, 2012). Objectified women can become subjects of both mechanistic dehumanization (Heflick and Goldenberg, 2009) and animalistic dehumanization (Vaes et al., 2011).

It is well known that, in the incongruity of voice (Taylor and Raadt, 2021; Fasoli et al., 2023), in the incongruity of gender role (Jetten et al., 2013), and incongruity of gender appearance (Gill, 1994; Rudman et al., 2012) can lead to negative reactions (Jetten et al., 2013). As gender incongruity increases, negative attitudes and discrimination can also intensify and women face greater penalties for gender incongruity than men who transgress gender roles (Fasoli and Hegarty, 2020). However, studies examining the impact of incongruity between voice tone, appearance and occupation on dehumanization are limited. Therefore, the main aim of the study is to investigate the influence of voice tone, in conjunction with the interaction between occupation and gender appearance incongruity, on the dehumanization of women.

### Context and hypothesis

Gender studies in Turkey have focused on the differences in roles between women and men in social, family, and work life (Dökmen, 2004; Sancar, 2011). In Turkey, the lack of equality laws, insufficient supportive political discourse, and organizational preparedness suggest that equality has not been achieved at the societal and organizational levels (Küskü et al., 2021; Kusku et al., 2022; Özbilgin et al., 2023). It is evident that women face inequalities in terms of education, employment, and representation, according to data from the Turkish Statistical Institute (2023). The Turkish labour market is dominated by middle-aged, Sunni Muslim, Turkish ethnicity, non-disabled heterosexual male workers (Göregenli et al., 2019). While typical workers dominate the labour market, ethnic minorities (Kurds), religious minorities, LGBTQ+ individuals, people with disabilities, immigrants from various countries, refugees, and women face low wages and limited job security in the Turkish labour market (Kusku et al., 2022).

Societal expectations regarding women and discrimination in the workplace serve as obstacles for women to reach managerial positions, causing a decrease in the number of women in managerial positions in the public sector (Bingöl et al., 2011). This contributes to high gender discrimination levels (Karatepe and Arıbaş, 2017, p. 7) in Turkey. Consistent with traditional gender roles, occupations such as teaching, medicine, and pharmacy, which are based on compassionate care and nurturing, are considered suitable pursuits for women, while professions like architecture, engineering, and management are not (Özkişi, 2012). It is deemed appropriate for women to work in jobs characterized by nurturing qualities and services, such as teaching, nursing, and flight attending, whereas men are expected to work in occupations requiring more independence, power, and leadership, such as engineering, contracting, management, and politics (Avcı et al., 2019).

Based on societal structures and related research, it can be said that the binary gender system that can be defined as the classification of individuals based on the sex characteristics assigned at birth, through social systems and cultural beliefs (Hyde et al., 2019; Morgenroth et al., 2021), is still more prevalent in Turkey (Köylü, 2016). The notion that men and women have different natures has been accepted, and men are expected not to behave, dress, laugh in feminine ways, or engage in women’s tasks (Gezici Yalçin and Tanriverdi, 2018). These differences have also been reflected in physical appearance and clothing choices, with clothing preferences becoming a significant reflection of gender norms.

As evident from the information provided above, gender inequalities persist in many areas of Turkey. Changing living conditions and cultural structures have the potential to reshape the meaning and content of gender. Especially with the influence of social media, femininity and masculinity have taken on new forms. What was once attributed to women can now be attributed to men, and vice versa (Gezici Yalçin and Tanriverdi, 2020). However, deviations from traditional masculinity can still lead to negative reactions (Gezici Yalçin and Tanriverdi, 2018).

#### Hypothesis

Gender stereotypes are associated with the phenomenon of dehumanization because they are formed through the socialization process. This is because traditional gender norms of masculinity and femininity, are also learned through social learning processes (Hemsing and Greaves, 2020). Because of these norms vary for men and women (Eagly, 1987; Fiske et al., 2007), individuals who do not conform to these norms may face negative reactions. For example, men who pursue professions considered feminine by society are subjected to ridicule (Isacco and Morse, 2015), while women who pursue professions considered masculine face scorn and discrimination (Jetten et al., 2013). Traditionally, women are expected to take on caregiving or emotional support roles within the family, while men are directed more towards outward, leadership roles (Eagly and Sczesny, 2019). So that we expect that occupation type will have a significant effect on the both mechanistic and animalistic dehumanization of women.

*Hypotheses 1a*: Women in occupations incongruent with gender expectation will have higher scores of mechanistic dehumanization than women in occupation congruent with gender expectation.

*Hypotheses 1b*: Women in occupations incongruent with female expectation will have higher scores of animalistic dehumanization than women in occupations congruent with female expectation.

Gender expectation about appearance also vary for men and women. In this regard men are often expected to have masculine appearance while women are expected to have feminine appearance (Connel, 1998, p. 109; Koenig, 2018; Van Grootel et al., 2018). However, individuals who exhibit behaviors not aligned with gender expectations face negative attitudes (Fiske and Stevens, 1993; Gill, 1994; Eagly and Wood, 2012; Rudman et al., 2012), research have found that gender expression has an effect on the mechanistic dehumanization. It was indicated that human nature characteristics are attributed more to feminine individuals compared to masculine individuals, regardless of sex (Diekman and Goodfriend, 2006; Heflick et al., 2011). Additionally, women who do not conform to gender expectations, based on gender expression, have higher scores of animalistic dehumanization compared to men who do conform (Tanriverdi and Gezici Yalçın, 2022). Therefore, we formulated hypotheses 2.

*Hypotheses 2a*: Women with appearances incongruent with gender expectation will have higher scores of mechanistic dehumanization than those with appearances congruent with gender expectation.

*Hypotheses 2b*: Women with appearances incongruent with gender expectation will have higher scores of animalistic dehumanization than those with appearances congruent with gender expectation.

Research about the impact of voice tone in impression formation are still inconclusive (Krahé and Papakonstantinou, 2020). In one study it is has been found that lower-pitched female voices were rated as more competent but less warm compared to higher-pitched (Oleszkiewicz et al., 2016). In another study both men and women with lower-pitched voices were perceived as more dominant and trustworthy (Borkowska and Pawlowski, 2011). However, some other studies imply that individuals with a tone of voice that is incongruent with their gender expectation may face negative reactions (Fuertes et al., 2011; Nelson et al., 2016; Fasoli et al., 2023). So that we formulated hypotheses 3.

*Hypotheses 3a*: Women with a tone of voice that is incongruent with gender expectations will have higher scores of mechanistic dehumanization than women with a tone of voice that is congruent with gender expectations.

*Hypotheses 3b*: Women with a tone of voice that is incongruent with gender expectation will have higher scores of animalistic dehumanization than women with a tone of voice that is congruent with gender expectation.

Visual and auditory cues can interact in the perception of gender (Smith et al., 2007) and influence social perception (Belin et al., 2004). As gender incongruity increases, negative attitudes and discrimination can also intensify. For instance, among women who identify as lesbians, those with a more masculine voice tone have been found to face more discrimination than men who identify as gay (Fasoli and Hegarty, 2020). Another study indicated that women who do not conform to gender expectations, based on gender expression, have higher scores of animalistic dehumanization compared to men who do conform and women who do not conform have higher scores of mechanistic dehumanization compared to women who do conform (Tanriverdi and Gezici Yalçın, 2022). Furthermore, individuals are expected to conform to gender roles stereotypically, and deviations from these roles can lead to negative reactions (Jetten et al., 2013). In addition, research suggests that although the perception of women’s competence is on the rise, women are still expected to uphold their feminine characteristics to counteract negative attitudes and reactions (Koenig, 2018). For all these reasons, we formulated hypothesis 4: the interaction effect of voice tone, appearance and occupation will be significant.

*Hypotheses 4a*: Women who are incongruent with gender expectations in terms of a greater number of variables (e.g., tone of voice, occupation, and appearance) are more likely to experience greater degrees of mechanistic dehumanization compared to those who are incongruent with gender expectations in fewer variables (e.g., occupation and appearance).

*Hypotheses 4b*: Women who are incongruent with gender expectations in terms of a greater number of variables (e.g., tone of voice, occupation, and appearance) are more likely to experience greater degrees of animalistic dehumanization compared to those who are incongruent with gender expectations in fewer variables (e.g., occupation and appearance).

However, we anticipate nuanced effects within each gender-related factor, such that the interaction between voice tone, appearance, and work will lead to differential levels of dehumanization depending on the specific combination of factors present.

## Method

### Participants and study design

Prior to data collection, a power analysis was conducted using G*Power (see Faul et al., 2007), which determined that 240 participants would be sufficient for a power of 0.95 and a high effect size (ƒ = 0.80). According to Hyde (2005), effect size is an important factor in indicating the statistical significance of differences between groups. The author interprets a value of *d* ≤ 0.10 as insignificant. Therefore, in the current study, efforts have been made to maintain a high effect. In this study, a total of 255 university students who were pursuing undergraduate or graduate education in different departments of Zonguldak Bülent Ecevit University were reached. Before proceeding to basic analyses, it was checked whether there was any missing data, and no missing data were detected. One participant had ranked items in descending order (5, 4, 3, 2, 1), and 2 participants had responded in ascending order (1, 2, 3, 4, 5). Generally, participants of this kind typically mark items without reading them. Therefore, these participants were excluded from the dataset. Subsequently, responses to manipulation control questions were examined. It was observed that 1 participant had answered the first question incorrectly, 6 participants answered the second question incorrectly, and 1 participant had answered all three questions incorrectly. Incorrectly answering manipulation control questions indicates that participants are not paying attention to manipulation, especially to occupation type. Therefore, it cannot be known whether they answered the scale questions based on appearance, tone of voice, or profession type. However, participants should answer considering all three variables. Therefore, these participants were also excluded from the dataset. The analysis continued with the data of the remaining 244 participants. The gender distribution of these participants was 125 females and 119 males, as reported in self-report forms. Participants’ ages ranged from 18 to 38 years (Mean_age_ = 21.87; SD = 2.47). A convenient sampling technique was applied to reach participants studying in various departments of their faculties, but randomization was carried out to assign participants to conditions. The study utilized a 2 (occupation: masculine, feminine) × 2 (gender appearance: masculine, feminine) × 2 (voice tone: masculine, feminine) between-subjects factorial design. The design of the research and the distribution of participants across conditions are presented in Table 1.

**Table 1:** Study design and sample distribution (*N* = 244).

Voice tone				
Work	Masculine		Feminine	
	Masculine	Feminine	Masculine	Feminine
*Appearance*				
Masculine	(*n* = 30)	(*n* = 32)	(*n* = 31)	(*n* = 31)
Feminine	(*n* = 30)	(*n* = 30)	(*n* = 30)	(*n* = 30)

### Data collection and measurement tools

#### Demographic information form

The demographic information form was designed to collect information such as age, gender, departments, and class levels of the participants in order to describe their demographic characteristics.

#### Dehumanization measurement

Studies have used human nature and human uniqueness characteristics to measure two forms of dehumanization (Haslam et al., 2005; Bain et al., 2009). The characteristics of human nature are common in society, universal across cultures, deeply rooted in humans and related to emotions, and are also formed at an early age in terms of development, while the characteristics of human uniqueness are formed later, are observed less frequently, and have relatively lower universality (Haslam, 2006). On the other hand, some of these characteristics (e.g., broad-minded, humble, polite, thorough) strongly distinguish humans from animal but lowly from machines; some of them (e.g., active, curious, friendly, fun-loving) strongly distinguish humans from machines but lowly from animals; and some of them (e.g., high-strung, insecure, irresponsible, reserved) strongly can distinguish humans from both animals and machines (Haslam et al., 2005). Therefore, we could not find a reliable and valid scale of the two forms of dehumanization in the literature. So that, based on the preview studies (e.g., Haslam et al., 2005; Bain et al., 2009), we used certain human characteristics such as “civilized,” “fair,” “logical,” and “honest” to measure the dimension of animalistic dehumanization, and others such as “sincere,” “warm,” “social,” “cheerful,” and “friendly” to measure the mechanistic dimension, in this study.

In the literature, most rating scales for attitude and opinion measures typically contain either five or seven response categories. Some researchers have suggested that reliability is maximized with seven-point scales (e.g., Finn, 1972; Ramsay, 1973). However, others have reported higher reliabilities for five-point scales (e.g., Jenkins and Taber, 1977; McKelvie, 1978). Additionally, research in cognitive psychology suggests that increasing the number of response options can lead to respondent confusion and increased cognitive burden (Cook et al., 2014). Thus, we rated the Likert-type scale ranging from 1 (Not at all appropriate) to 5 (Completely appropriate), aiming to minimize respondent fatigue and enhance the clarity and simplicity of the scale, thereby reducing the likelihood of response errors (Preston and Colman, 2000). Scores obtained from each attribute were reverse-coded, and to differentiate them into the two dimensions, their validity and reliability were tested. Higher average scores from each dimension indicate higher dehumanization in the respective dimension.

### Procedure

Before commencing the study, ethical approval was obtained from the Human Research Ethics Committee of Zonguldak Bülent Ecevit University, dated March 29, 2023, under Senate Resolution 2014/08-13 (Protocol no: 93). To conduct the research, a laboratory was prepared. For the manipulation of voice tone, two female speakers (one with a feminine voice and one with a masculine voice) were selected from the Conservatory and Opera Department based on the opinions of four professional academics specializing in voice tone within the opera department. Then, in a quiet environment, using a plain-speaking style (e.g., accent-free, simple, and free from slang), the speakers recorded the standardized text (utilizing both feminine and masculine voices) containing the predetermined feminine and masculine professions. For the profession manipulation, engineering, considered masculine, and kindergarten teaching, considered feminine based on literature (Eagly, 2013) and studies in Turkey (see Özkişi, 2012; Avcı et al., 2019) were chosen. The name “Deniz,” a non-binary gender name, was used for the target person in the vignette. Women with suitable voice tones read a text indicating that they belong to a profession, and these recordings were made. To manipulate appearance, lively colors, dresses, and long hair were preferred for a feminine appearance, while dark colors, suits, and short hair were chosen for a masculine appearance, based on literature (Ridgeway, 2014; Köylü, 2016). A photo shoot was conducted with a permitted model wearing these clothes. In Photoshop, hair lengths (short and long) were adjusted, and a single background (white) was used to prevent distracting elements. During data collection, participants were presented with this vignette in a laboratory setting while listening to it in either a masculine or feminine voice tone through a computer, and they were simultaneously shown a darkened image of a woman with either a masculine or feminine appearance. To ascertain participants’ attentiveness to the manipulations, three questions were posed during data collection, focusing on Ms. Deniz’s occupation, employment status, and level of education. Participants, admitted to the laboratory in pairs, were informed about the research and provided informed consent forms. Participants were instructed to select a number between 1 and 8, facilitating random assignment to conditions. Following this, audio recordings corresponding to their chosen condition were played through headphones, and a 5-point Likert scale was employed for dehumanization measurement based on the condition they listened to. Four experts in the field were consulted for manipulation control, and feedback was obtained from all experts confirming the successful implementation of the manipulation. In addition, participants were asked with two 5-point Likert-type questions ranging from 1 (Completely appropriate) to 5 (Not at all appropriate) to what extent they found the target person masculine or feminine for each (Voice, Work, Appearance) manipulation condition. One of these questions was reverse coded and whether there was a significant difference between masculine and feminine conditions was tested with independent samples t-test analysis. Data were subjected to MANCOVA (multivariate analysis of covariance) analysis.

## Results

For the data analysis of this study, SPSS 26.0 statistical analysis software was utilized. Independent samples t-test analysis used for manipulations control, Exploratory Factor Analysis (EFA) was initially conducted to determine the validity of the measurement instrument used, and Cronbach’s Alpha coefficient was calculated to determine its reliability. Skewness and kurtosis values of the dependent variables were examined to test whether the data followed a normal distribution. Subsequently, a multivariate analysis of covariance (MANCOVA) was conducted to test the effects of independent variables on dependent variables, with participant gender included as a control variable.

### Results of independent samples *t*-test analysis

According to independent samples t-test analysis, the difference between the masculine voice (*M* = 3.93, SD = 1.71) and feminine voice (*M* = 2.33, SD = 0.75) condition was significant [*t*(235) = 9.34; *p* < 0.000, Cohen’s *d* = 0.51]. The results of Levene’s test for equality of variances showed violations, *p* = 0.000. This mean that the target person in masculine voice condition was perceived as having more masculine voice than the target person in feminine voice condition. The difference between the masculine work (*M* = 5.60, SD = 1.54) and feminine work (*M* = 3.61, SD = 1.74) condition was also significant [*t*(235) = 9.26; *p* < 0.000, Cohen’s *d* = 0.51]. The results of Levene’s test for equality of variances showed violations, *p* = 0.004. This mean that the masculine work condition was perceived more masculine than feminine work condition. The difference between the masculine appearance (*M* = 6.57, SD = 2.20) and feminine appearance (*M* = 2.60, SD = 1.33) condition was also significant [*t*(235) = 16.77; *p* < 0.000, Cohen’s *d* = 0.73]. The results of Levene’s test for equality of variances showed violations, *p* = 0.000. This mean that the target person in masculine appearance condition was perceived more masculine than the target person in feminine appearance condition. In overall, based on these results, it can be said that manipulation was achieved in all three conditions.

### Findings of exploratory factor analysis and reliability analysis

In the factor analysis conducted on the 25 items in the dehumanization scale, we attempted to obtain a two-factor structure. A pre-determined value for the number of factors was not used; instead, a method is suggested in the literature where factors with eigenvalues greater than 1 or equal to 1 are considered significant (Büyüköztürk, 2002). According to the analysis results, 8 items (10, 11, 16, 17, 19, 23, 24, 25) disrupt the two-factor structure. When these items were removed, the two-factor structure of the scale became more evident. Upon examination of the factor structure, it was observed that the factor loadings and cumulative values of all items were above 0.40. Consequently, the first factor with an eigenvalue of 7.83 explained a variance of 46.07%, while the second factor with an eigenvalue of 1.96 explained a variance of 11.58%. The total variance explained by these two factors was 57.66%. Considering previous studies (e.g., Haslam et al., 2005; Bain et al., 2009) the first factor, “Animalistic Dehumanization,” consisted of 12 items with a reliability coefficient of *α* = 0.91. The second factor, “Mechanistic Dehumanization,” included 5 items, and the reliability coefficient was *α* = 0.90. The total reliability coefficient of the scale was calculated as *α* = 0.92 (see Table 2). These results suggest that dehumanization can be measured reliably and validly in two dimensions.

**Table 2:** Results of EFA and reliability analysis for the dehumanization scale.

Traits	Communalities	Factor load	
Item 6: fair	0.556	0.856	
Item1: trustworthy	0.403	0.802	
Item 5: honest	0.436	0.792	
Item 8: moral	0.542	0.765	
Item 22: conscientious	0.612	0.737	
Item 7: resolute	0.672	0.703	
Item 2: logical	0.455	0.677	
Item 9: creative	0.662	0.663	
Item 4: humble	0.458	0.603	
Item 18: able to plan	0.639	0.587	
Item 3: broadminded	0.769	0.551	
Item 20: capable of feeling guilt	0.844	0.532	
Item 14: friendly	0.761		0.947
Item 15: cheerful	0.429		0.893
Item 13: warm	0.574		0.859
Item 12: sincere	0.419		0.772
Item 21: social	0.572		0.664
Cronbach’s alpha	0.92	0.91	0.90

### Findings of multivariate analysis of covariance

This study aimed to investigate the effects of voice, occupation, appearance, and interaction effects of these variables on dehumanization. Descriptive statistics were calculated to provide an overview of the dehumanization scores across different combinations of voice, occupation, and appearance (see Table 3). The mean mechanistic and animalistic dehumanization scores varied across these conditions, suggesting potential differences in how participants perceived individuals in each scenario. The overall model significantly predicted dehumanization levels for both mechanistic (*F*_(8, 234)_ = 5.541, *p* < 0.001, *η*^2^ = 0.159) and animalistic (*F*_(8, 234)_ = 2.368, *p* = 0.002, *η*^2^ = 0.075) dehumanization.

**Table 3:** Descriptive statistics.

Variables	Voice	Appearance	Occupation	Mean	Std. Dev.	*N*
Mechanistic	Masculine	Masculine	Masculine	12.9000	5.07428	30
			Feminine	8.9667	3.89945	30
			Total	10.9333	4.90543	60
		Feminine	Masculine	10.8438	4.00894	32
			Feminine	11.6333	4.08938	30
			Total	11.2258	4.03436	62
		Total	Masculine	11.8387	4.63484	62
			Feminine	10.3000	4.18350	60
			Total	11.0820	4.46768	122
	Feminine	Masculine	Masculine	10.8387	4.84491	31
			Feminine	7.8710	2.84888	31
			Total	9.3548	4.21588	62
		Feminine	Masculine	10.2667	3.86793	30
			Feminine	7.7000	3.67799	30
			Total	8.9833	3.95951	60
		Total	Masculine	10.5574	4.36472	61
			Feminine	7.7869	3.25635	61
			Total	9.1721	4.07916	122
	Total	Masculine	Masculine	11.8525	5.02605	61
			Feminine	8.4098	3.42236	61
			Total	10.1311	4.61752	122
		Feminine	Masculine	10.5645	3.91977	62
			Feminine	9.6667	4.33616	60
			Total	10.1230	4.13727	122
		Total	Masculine	11.2033	4.53033	123
			Feminine	9.0331	3.93686	121
			Total	10.1270	4.37495	244
Animalistic	Masculine	Masculine	Masculine	30.5333	8.01177	30
			Feminine	29.1000	9.98050	30
			Total	29.8167	9.00187	60
		Feminine	Masculine	30.3750	8.14684	32
			Feminine	30.0000	7.01230	30
			Total	30.1935	7.55925	62
		Total	Masculine	30.4516	8.01572	62
			Feminine	29.5500	8.56367	60
			Total	30.0082	8.26773	122
	Feminine	Masculine	Masculine	30.9032	8.72680	31
			Feminine	25.1613	6.71861	31
			Total	28.0323	8.24813	62
		Feminine	Masculine	29.7333	9.31048	30
			Feminine	24.9333	8.90538	30
			Total	27.3333	9.35127	60
		Total	Masculine	30.3279	8.96237	61
			Feminine	25.0492	7.80476	61
			Total	27.6885	8.77834	122
	Total	Masculine	Masculine	30.7213	8.31491	61
			Feminine	27.0984	8.64042	61
			Total	28.9098	8.63780	122
		Feminine	Masculine	30.0645	8.66285	62
			Feminine	27.4667	8.34727	60
			Total	28.7869	8.57368	122
		Total	Masculine	30.3902	8.46365	123
			Feminine	27.2810	8.46288	121
			Total	28.8484	8.58829	244

Tests of between-subjects effects were conducted to examine the significance of the main effects and interactions on mechanistic and animalistic dehumanization. Results demonstrated that occupation type had a significant main effect on mechanistic dehumanization (*F*_(1, 235)_ = 17.592, *p* < 0.000, *η*^2^ = 0.070) and animalistic dehumanization (*F*_(1, 235)_ = 8.45, *p* = 0.004, *η*^2^ = 0.035), indicating that women who have masculine occupations are more dehumanized than who have feminine occupations. Hypothesis 1a and Hypothesis 1b are supported. Voice tone had also a significant main effect on mechanistic dehumanization (*F*_(1, 235)_ = 13.532, *p* < 0.00, *η*^2^ = 0.54) and animalistic dehumanization (*F*_(1, 235)_ = 4.68, *p* = 0.041, *η*^2^ = 0.020). Hypothesis 3a and Hypothesis 3b are supported. This suggests the women who have masculine voice are more dehumanized than who have feminine voice tone. This indicates that participants’ perception of mechanistic and animalistic dehumanization significantly varied based on the voice tone and work occupation presented in the stimuli. However, the main effect of appearance, and participants’ gender did not yield significance for either mechanistic or animalistic dehumanization (*p* > 0.05). Hypothesis 2 rejected. The interaction between appearance and occupation had a significant effect on mechanistic dehumanization (*F*_(1, 235)_ = 6.010, *p* = 0.01, *η*^2^ = 0.025) but not on animalistic dehumanization (*p* > 0.05). Pairwise comparison indicated that engineer women who adopt masculine appearances are more dehumanized (Mean = 11.87, SE = 0.74) compare to engineer women with feminine appearance [Mean = 10.57, SE = 0.73, *p* < 0.001, 95% CI (2.018, 4.930)]. This implies that the effect of appearance on the mechanistic dehumanization of women depended to occupation. The interaction effect of voice and occupation and voice and appearance were not significant (p > 0.05). The three-way interaction between voice, appearance, and occupation was also significant for mechanistic dehumanization (*F*_(1, 235)_ = 4.279, *p* = 0.04, *η*^2^ = 0.018) but not for animalistic dehumanization (*p* > 0.05). Hypothesis 4a is supported but hypothesis 4b is rejected. This indicates that the impact of voice tone on de mechanistic dehumanization in the workplace depend on appearance type. It also suggests that the more gender incongruity the more mechanistic dehumanization of women.

**Figure 1:**
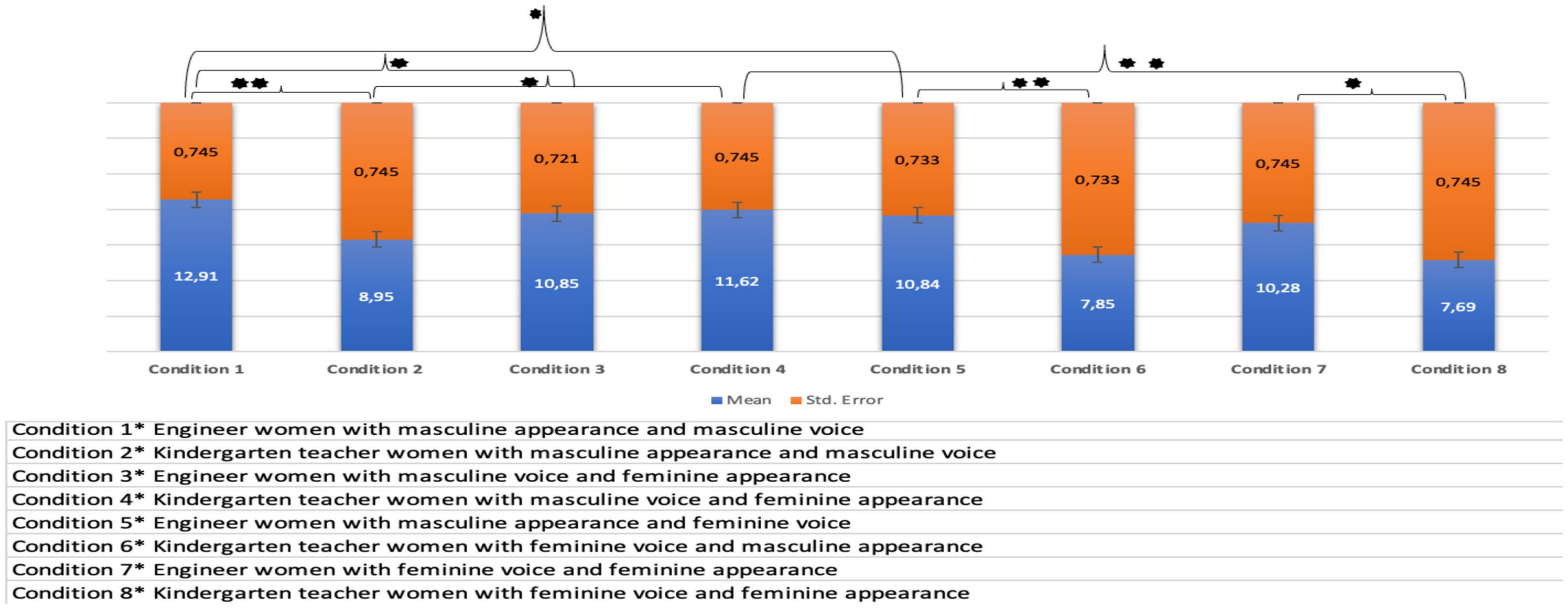
Pairwise comparisons of between subject.

We used simple effect analysis to compare the levels of mechanistic dehumanization between different conditions (see Figure 1). The results showed that participants’ scores for mechanistic dehumanization in Condition 1 (engineer women with masculine appearance and masculine voice: Mean = 12.91, SE = 0.74) were significantly higher than those in Condition 2 [kindergarten teacher women with masculine appearance and masculine voice: Mean = 8.95, SE = 0.74, *p* < 0.001, 95% CI (1.885, 6.013)]. This indicates that Engineer women with masculine voices and appearances face more dehumanization than kindergarten teacher women with similar voice tones and appearances. Participants’ scores in Condition 1 (engineer women with masculine appearance and masculine voice: Mean = 12.91, SE = 0.74) were significantly higher than those in Condition 3 [engineer women with feminine appearance and masculine voice: Mean = 10.85, SE = 0.72, *p* < 0.005, 95% CI (0.015, 4.10)]. This suggests that engineer women with masculine appearances and voices are more dehumanized than those with masculine voices but feminine appearances. Participants’ scores in Condition 1 (engineer women with masculine appearance and masculine voice: Mean = 12.91, SE = 0.74) were significantly higher than those in Condition 5 [engineer women with feminine voice and masculine appearance: Mean = 10.84, SE = 0.73, *p* < 0.005, 95% CI (0.015, 4.13)]. This means that engineer women with masculine voices and appearances face more dehumanization compared to those with feminine voices but masculine appearances. Participants’ scores in Condition 4 (kindergarten teacher women with feminine appearance and masculine voice: Mean = 11.62, SE = 0.73) were significantly higher than those in Condition 2 [kindergarten teacher women with masculine appearance and masculine voice: Mean = 8.95, SE = 0.74, *p* < 0.005, 95% CI (0.54, 4.74)]. This indicates that kindergarten teacher women with masculine voices and appearances face more dehumanization than those with feminine appearances but masculine voices. Participants’ scores in Condition 4 (kindergarten teacher women with feminine appearance and masculine voice: 11.62, SE = 0.73) were significantly higher than those in Condition 8 [kindergarten teacher women with feminine voice and feminine appearance: Mean = 7.69, SE = 0.74, *p* < 0.001, 95% CI (1.85, 6.00)]. This means that kindergarten teacher women with masculine voices and feminine appearances are more dehumanized than those with feminine voices and appearances. Participants’ scores in Condition 5 (engineer women with feminine voice and masculine appearance: Mean = 10.84, SE = 0.73) were significantly higher than those in Condition 6 [kindergarten teacher women with feminine voice and masculine appearance: Mean = 7.85, SE = 0.73, *p* < 0.001, 95% CI (0.94, 5.03)]. This indicates that engineer women with a masculine appearance and a feminine voice experience more dehumanization than kindergarten teacher women with the same characteristics. Participants’ scores in Condition 7 (engineer women with feminine voice and feminine appearance: Mean = 10.28, SE = 0.74) were significantly higher than those in Condition 8 [kindergarten teacher women with feminine voice and feminine appearance: Mean = 7.69, SE = 0.74, *p* < 0.005, 95% CI (0.51, 4.66)]. This suggests that engineer women with feminine voices and appearances may face more dehumanization than kindergarten teacher women with similar voices and appearances.

## Discussion

This study aimed to investigate the impact of voice tone, appearance and occupation type on mechanistic and animalistic dehumanization of women. The findings emphasize the significant influence of societal norms and expectations on how individuals are perceived, highlighting that woman deviating from traditional gender roles are at risk of dehumanization. As expected, hypotheses 1a and hypotheses 1b, emphasizing the impact of occupation type on dehumanization of women were supported. This result appears consistent with studies indicating that gender nonconformity is not well-received (Jetten et al., 2013; Isacco and Morse, 2015), that traditionally, women are directed towards lower-paying and lower-status jobs (Fiske and Stevens, 1993), and that in order to avoid these negative consequences, people performed their gender expectations that leading to the perpetuation of gender inequalities (Eagly, 1987; Butler, 1990).

Hypotheses 3a and 3b, which emphasize the impact of voice tone on dehumanization, were supported. Individuals with masculine voices and masculine occupations experienced both mechanistic and animalistic dehumanization. These results underscore the role of perception and bias associated with voice characteristics suggesting that individuals who do not conform to a gender-appropriate voice tone are likened to both objects and animals. Previous studies have underscored the positive impact of a lower-pitched voice, particularly in relation to the perception of competence (Borkowska and Pawlowski, 2011; Oleszkiewicz et al., 2016). The findings of the current study suggest that women who exhibit incongruence with their voice tone may be perceived as less human in terms of attributes such as friendliness, cheerfulness, warmth, and sincerity, as well as in terms of fairness, honesty, morality, conscientiousness, resolution, creativity, and open-mindedness. The findings more align with previous research indicating that incongruence with tone of voice can lead to negative reactions (Fuertes et al., 2011; Nelson et al., 2016; Fasoli et al., 2023).

Hypothesis 2a and 2b which emphasize the impact of appearance on dehumanization of women was not supported. However, the interaction effect of appearance and occupation was significant on the mechanistic dehumanization. This statement means that in the case of kindergarten teachers (congruence with occupation expectation), whether they adopt a masculine appearance or a feminine appearance does not significantly affect the level of mechanistic dehumanization they experience. However, for engineer women (incongruence with occupation expectation), there is a significant difference in mechanistic dehumanization between those who adopt a masculine appearance and those who adopt a feminine appearance. In other words, for engineers, the choice of appearance has a noticeable impact on the level of dehumanization they face, whereas for kindergarten teachers, appearance seems to have less influence on dehumanization. In addition, hypothesis 4a, regarding the interactive effects of gender role, gender appearance, and voice tone, was supported as well. This hypothesis suggested that the level of mechanistic dehumanization towards women would vary based on different combinations of voice tone, gender role, and gender appearance. The findings reveal that the combination of these variables creates differences in mechanical dehumanization. For example, women who are incongruent in all three variables are exposed to more mechanical dehumanization compared to those who are incongruent in appearance but congruent in voice tone or those who are congruent in appearance but incongruent in voice tone and occupation. This result suggests that women who deviate from traditional gender norms across multiple variables may experience higher levels of dehumanization. In other words, when women exhibit incongruence with societal expectations in terms of their appearance, voice tone, and occupation, they are more likely to face dehumanizing attitudes and treatment, consistent with preview study (Fasoli and Hegarty, 2020).

These results resonate with objectification theory, which posits that individuals, particularly women, are often objectified based on their appearance and perceived adherence to societal beauty standards (Fredrickson and Roberts, 1997). In this case, women with masculine voices and masculine appearance, and women working in masculine occupation may be objectified, as their characteristics deviates from traditional gender norms, leading to perceptions of them as less human (Fuertes et al., 2011; Fasoli et al., 2023).

On the other hand, hypothesis 4b, testing the interaction effect of the three variables on animalistic dehumanization, was rejected. While voice tone and occupation had significant effects on animalistic dehumanization, their interaction with appearance did not yield significant results. This means that both kindergarten teachers and female engineers, whether they adopt a masculine or feminine appearance or have a masculine or feminine voice, do not significantly affect the level of animalistic dehumanization they experience. Previews studies implied that gender expression in terms masculinity and femininity, is more closely associated with mechanistic dehumanization (Diekman and Goodfriend, 2006; Heflick et al., 2011; MacInnis and Hodson, 2012; Tanriverdi and Gezici Yalçın, 2022). This is also possible explanation of why our hypothesis 2b was not supported. The current study appears consistent with these studies. While voice tone does indeed have an impact on the dehumanization of women, it appears that this effect does not vary significantly based on occupation type. This suggests that regardless of the type of occupation, women may face similar levels of dehumanization based on their voice tone.

Additionally, the gender of the participants was found to have no significant effect on dehumanization towards women. This suggests that individual characteristics do not heavily influence dehumanization of women. Previous studies have also indicated that there is no significant difference in attributing human traits based on participant gender (Bain et al., 2009; Vaes et al., 2011).

One of the major contributions of this study is shedding light on how seemingly minor details, such as voice tone can play a significant role in dehumanizing women. Furthermore, this study contributes to a better understanding of the effects of occupation and societal norms in the workplace. It also offers insights into how various combinations of occupation, gender appearance, and voice tone can affect dehumanization of women, subsequently influencing their experiences of negative attitudes, behaviors, and discrimination. Overall, this study delves deep into the relationship between gender congruence or incongruence in occupation, gender appearance, and voice tone and dehumanization, helping us comprehend how gender norms and societal expectations impact dehumanization of women. Social and organizational interventions which target gender equality need to consider the impact of gender norms on dehumanization of women through processes of evaluation of occupation, appearance and voice tone. Recognizing varied manifestations of dehumanization of women, in turn, can contribute to design and development of gender equality interventions that combat dehumanization, promoting fairer and more egalitarian work environments.

## Limitations and recommendations

One of the primary limitations of this study pertains to the manipulation of voice tone, which relied on information provided by experts in the Conservatory and Opera Department as well as professionals in gender literature. Specifically, the categorization of masculine and feminine voices was determined based on the expertise of these professionals rather than through a systematic analysis of frequency and formant characteristics. Individuals with lower-frequency (within 100–120 Hz) voices are often perceived as more masculine while those with higher-frequency (200–220 Hz) voices tend to be perceived as more feminine (Pisanski and Bryant, 2019). Therefore, future research endeavors could benefit from conducting pilot studies aimed at analyzing the frequency and formant characteristics of voice in order to more accurately determine its masculinity or femininity.

Another limitation is that in this study we focused only on the dehumanization of women. Considering men in future research would enable us to explore whether similar principles of gender congruence and stereotypes apply, albeit within potentially different societal expectations and norms. Just as women may face dehumanization when deviating from traditional gender roles, men who display nonconforming traits or behaviors may also encounter negative reactions and biases. However, because of femineity related to human nature characteristic (Diekman and Goodfriend, 2006; Heflick et al., 2011) display nonconforming expectation may increase the attribution of human nature characteristics to men. By investigating these dynamics among men, we can gain a more comprehensive understanding of how gender norms influence perceptions of dehumanization in the workplace. Moreover however, research found that sexual orientation is related to mechanistic dehumanization, based on masculinity and femineity (MacInnis and Hodson, 2012; Vaughn et al., 2017), future research would also can test the interaction effect of voice tone, appearance, occupation type and sexual orientation.

Furthermore, in light of the findings of this study, there arises a necessity for more comprehensive research to investigate the effects of various combinations of occupation, gender appearance, and voice tone on animalistic dehumanization. It is evident that these variables interact to different extents in influencing mechanistic dehumanization, emphasizing the importance of conducting detailed investigations in this area. Moreover, other factors such as personality traits, cultural influences, and situational contexts were not fully examined. Future studies could incorporate these additional variables to gain a more nuanced understanding of the complexities surrounding dehumanization processes.

Lastly, the study utilized self-report measures to assess dehumanization, which may be subject to biases and social desirability effects. Incorporating objective measures or observational methods could enhance the validity and reliability of the findings.

## Conclusion

This study highlights the persistent influence of gender expectations on dehumanization. The results demonstrate that various factors can significantly affect both mechanistic and animalistic dehumanization towards women. Voice tone and occupation type emerge as critical factors in the dehumanization of women. Having a masculine voice is associated with higher dehumanization of women. Similarly, women in professions challenging traditional occupations may also be more vulnerable to dehumanization. Moreover, the interaction of these variables can influence mechanistic dehumanization differently, revealing that the harmony or incongruity of voice tone, physical appearance, and occupation can impact mechanistic dehumanization. This suggests resistance to changing societal expectations, as traditionally masculine qualities, when displayed by women, may lead to their dehumanization. This finding aligns with previous research indicating that masculinity is highly valued and that women displaying masculine qualities can be dehumanized (Nutt, 2005; Boratav et al., 2017). Further research is needed to fully understand the complexity of the interaction of these factors, especially in relation to animalistic dehumanization.

## Data availability statement

The raw data supporting the conclusions of this article will be made available by the authors, without undue reservation.

## Ethics statement

The studies involving humans were approved by Human Research Ethics Committee of Zonguldak Bülent Ecevit University, dated March 29, 2023, under Senate Resolution 2014/08-13 (Protocol no: 93). The studies were conducted in accordance with the local legislation and institutional requirements. The participants provided their written informed consent to participate in this study.

## Author contributions

VT: Formal analysis, Methodology, Writing – original draft. AY: Writing – original draft. ET: Writing – original draft. MO: Writing – review & editing.
